# ESD-aid surgery as a new treatment strategy for duodenal adenoma

**DOI:** 10.1186/s13104-022-05922-7

**Published:** 2022-02-10

**Authors:** Tomotaka Okubo, Ryo Ogawa, Shuhei Ueno, Sunao Ito, Shunsuke Hayakawa, Hiroyuki Sagawa, Tatsuya Tanaka, Hiroki Takahashi, Yoichi Matsuo, Takaya Shimura, Hiromi Kataoka, Shuji Takiguchi

**Affiliations:** 1 Department of Gastroenterological Surgery, Nagoya City University Graduate School of Medical Sciences, 1 Kawasumi, Mizuho-cho, Mizuho-ku, Nagoya, 467-8601 Japan; 2 Department of Gastroenterology and Metabolism, Nagoya City University Graduate School of Medical Sciences, 1 Kawasumi, Mizuho-cho, Mizuho-ku, Nagoya, 467-8601 Japan

**Keywords:** Duodenal adenoma, ESD, Surgery

## Abstract

**Objective:**

The treatment for nonampullary duodenal adenoma remains to have no consensus and established methods. Although endoscopic treatment is minimally invasive, it was reported to cause delayed perforation in more than 20% of cases. For adenomas in the duodenum, we performed endoscopic submucosal dissection (ESD)-aid surgery, which is a procedure to prophylactically suture the seromuscular structure of the duodenum after ESD. In this procedure, we did not perform Kocher mobilization prior to ESD to facilitate endoscopic resection and full-thickness resection to prevent spread of the tumor and infection to the abdominal cavity. The duodenal wall was reinforced in planes using a suture clip.

**Results:**

Of the 13 cases of duodenal adenoma that underwent ESD-aid surgery at our hospital between April 2018 and December 2020, 1 developed postoperative bleeding, but there was no late perforation. For duodenal adenomas, ESD-aid surgery was considered a safe and minimally invasive treatment.

## Introduction

In recent years, duodenal nonampullary adenomas have been increasingly detected because of the increasing frequency and improved accuracy of endoscopy. Although there have been multiple treatments for duodenal adenomas in the nonduodenal papilla, there had been no established therapy to come up with a consensus. Endoscopic treatment is less invasive but technically difficult and carries the risk of complications, such as delayed bleeding and perforation. In particular, delayed perforation has been reported to account for more than 20% of cases [1, 2]. On the other hand, surgical treatment is highly invasive.

Nowadays, laparoscopy and endoscopy cooperative surgery (LECS) has been reported as a treatment for duodenal adenoma. At our hospital, we treat cases of nonampullary duodenal adenoma with endoscopic submucosal dissection (ESD)-aid surgery, in which the duodenal wall is surgically sutured and reinforced after excision of the duodenal adenoma by ESD. In this study, we reported the utility and safety of ESD-aid surgery as a new concept of treatment for duodenal adenoma.

## Main text

### Methods

#### Study design

This retrospective study included patients who had been admitted for ESD-aid surgery from April 2018 to December 2020 at Nagoya City University Hospital in Japan. We performed ESD-aid surgery on tumors that were  ≥ 15 mm or those that were about 10 mm in size and judged to have a high risk for perforation. The records of these patients were retrospectively reviewed for clinical data, including patient characteristics, tumor location and size, operation time, blood loss, postoperative hospital days, and complications. Based on this, we investigated the safety and efficacy of ESD-aid surgery.

#### Operative technique

**Fig. 1:**
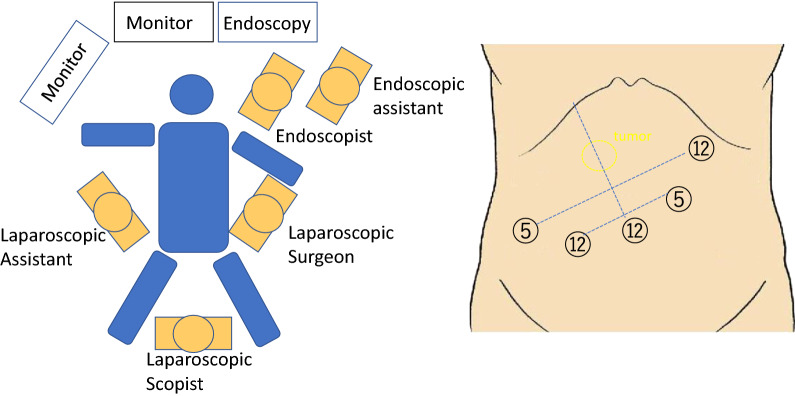
Deployment of operating room staff and trocar arrangement for ESD-aid surgery. A 12-mm trocar is placed into the navel as a camera port. Four trocars are placed on positions that are symmetrical to the line connecting the navel and the tumor. *ESD* endoscopic submucosal dissection

**Fig. 2:**
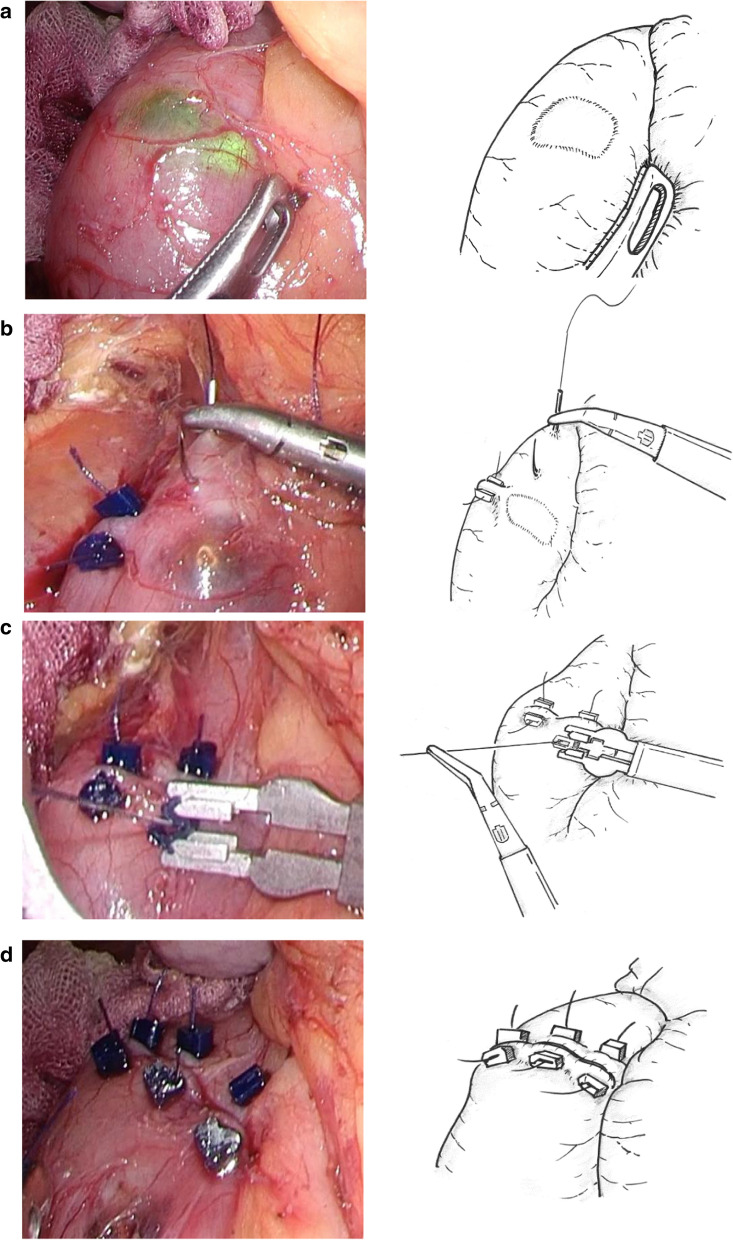
ESD-aid surgery for nonampullary duodenal adenoma. **a** The excised part of the duodenal tumor is confirmed laparoscopically. **b**, **c** The serosal muscle is attached to the duodenal wall using suture and suture clips to reinforce the thinned area. **d** Completion of the procedure. *ESD* endoscopic submucosal dissection

ESD-aid surgery was performed under general anesthesia, with the patient in the open leg position. The trocars were surgically placed in the abdomen. The first trocar was placed in the navel. Next, four trocars were placed in positions that were symmetrical to the line that connected the navel and the tumor (Fig. 1). The intestine was clipped 10–20 cm from the Treitz ligament in order to prevent air from flowing into the small intestine. Thereafter, the endoscopist performed ESD to resect the tumor, followed by reverting to a laparoscopic operation. After mobilizing the hepatic flexure of the colon, Kocher mobilization was performed from the second to the third part of the duodenum, depending on the tumor location. Tumor incision was confirmed laparoscopically and endoscopically. Using absorbent threads and suture clips (LAPRA-TY^®^ Johnson and Johnson Institute, New Brunswick, America), a seromuscular suture was applied to the duodenum along the long axis in order to draw the scar portion of the tumor closer (Fig. 2). The abdominal cavity was washed, and the absence of bleeding was confirmed before drain placement and wound closure.

## Results

During the period, there were 140 cases of duodenal adenoma resected endoscopically, and 13 patients of them were indicated for ESD-aid surgery. Of the patients, 11 were men and 2 were women. The age range of the patients was 47–82 years (median, 67 years).

The duodenal tumor location was on the second part in 9 cases and on the third part in 4 cases. The major diameter of the tumor ranged 15–40 mm (median, 25 mm).

The operation time was 210–534 min (median, 292 min); the endoscopic operation time was 72–342 min (median, 149 min); and blood loss was 6–72 mL (median, 37 mL).

Postoperative hospital stay was 9–18 days (median, 11 days), and Clavien–Dindo grade II postoperative bleeding was observed in one case.

The postoperative observation period was 4–31 months (median, 13 months). No delayed perforation or postoperative stenosis was observed. All pathologic findings showed adenoma, and no malignant tumors were observed (Table 1).

**Table 1:** Patient and tumor characteristics and surgical outcomes

Parameters	
Sex (male/female)	11/2
Age (years)	67 (47–82)
Location	
2nd	9
3rd	4
Size (mm)	25 (15–40)
Operation time (min)	292 (210–534)
ESD time (min)	129 (72–342)
Blood loss (mL)	37 (6–72)
Postoperative complications (n)	1 (postoperative bleeding)
Postoperative hospital stay (days)	11 (9–18)
Follow-up period (months)	13 (4–31)

## Discussion

Nonampullary duodenal adenoma, which is a rare disease that is often found during endoscopy screening [3], is considered to be at risk for carcinoma and is usually endoscopically resected at several institutions. However, endoscopic resection has been reported to have many highly severe adverse events, such as perforation and delayed perforation [1, 2]. On the other hand, with surgical treatment, accurate identification of the position and range of the tumor before surgery is often difficult and would require an intraoperative endoscope and clipping for marking. Pancreatoduodenectomy is highly invasive and may be an overtreatment. Although there are limited surgical options, such as transduodenal resection, partial thickness partial resection, duodenal resection, and segmentectomy [4–7], these are more invasive, compared with the cost of endoscopic treatment. Few reports have mentioned the application of a laparoscopic approach to duodenal reduction surgery. Thus, we developed a treatment method that had the advantages of both endoscopy and laparoscopy.

Hiki et al. first described LECS as a treatment for GIST [8]. In classical LECS, the gastrointestinal tract is incised to invert and resect the tumor, thereby, posing a risk for tumor dissemination. On the other hand, with ESD-aid surgery, ESD was performed in order to avoid puncture by endoscope operation and for resection of the adenoma. Total resection was not performed to prevent spread of the tumor and infection into the abdominal cavity. Irino et al. reported a case of laparoscopic reinforcement from the serosa side after ESD as a new method of LECS [9]. They use only sutures to reinforce the duodenal wall. After ESD, the wall of the duodenum is thin and easy to tear. Reinforcement of sutures alone can easily tear the duodenal wall. Therefore, we used both sutures and suture clips to reinforce the duodenal wall. With the use of the suture clip, the duodenal wall could be drawn in a planar direction, thereby, preventing tears and enabling safe reinforcement after ESD, even for a large tumor. In addition, suturing along the long axis can prevent postoperative stenosis.

We have evolved and standardized the procedure for duodenal adenoma as ESD-aid surgery, which we considered to encompass the concept of LECS. Resection of duodenal adenoma is mainly based on ESD, and ESD-aid surgery was a technique to supplement ESD.

There were no postoperative complications, except for one case of postoperative bleeding. No case of delayed perforation was observed. Based on this, we considered that ESD-aid surgery can sufficiently contribute to the prevention of postoperative perforation. The main disadvantage of ESD-aid surgery was the longer operation time, compared with the duration of ESD alone. However, there was no increase in the number of complications that may occur during the subsequent course after the prolonged operation time. Therefore, extension of the operation time may be acceptable. In these cases, the long-term prognosis of postoperative stenosis or recurrence could not be fully examined because of the short observation period; therefore, future follow-up would be necessary.

## Conclusions

Using the advantages of endoscopy and laparoscopy, ESD-aid surgery may be considered a safe and minimally invasive treatment for duodenal adenoma.

## Limitations

In this study, the number of cases is still small, and it is considered necessary to accumulate cases in the future. In addition, the observation period is short and it is necessary to consider the long-term prognosis.

## Data Availability

The datasets used and/or analyzed during the current study are available from the corresponding author on reasonable request.

## Abbreviations

ESD: Endoscopic submucosal dissection
LECS: Laparoscopy and endoscopy cooperative surgery
